# Soil microbiota enhance the population growth rate of a nitrogen-fixing herbaceous legume

**DOI:** 10.1093/aobpla/plaf012

**Published:** 2025-03-16

**Authors:** Satu Ramula, James D Blande, Aino Kalske

**Affiliations:** Department of Biology, University of Turku, 20014 Turku, Finland; Department of Environmental and Biological Sciences, University of Eastern Finland, Yliopistonranta 8, 70210 Kuopio, Finland; Department of Biology, University of Turku, 20014 Turku, Finland

**Keywords:** demography, integral projection model, invasive species, plant-microbe interaction, population growth rate, population model, volatiles

## Abstract

Soil microbiota can modify a plant’s growth and chemistry, with consequences for plant population persistence. Therefore, an approach that considers the entire life cycle of a given plant species may be necessary for quantifying the net effect of soil microbiota on longer-lived plants. Here, we investigated the effects of soil microbiota on the fitness-related traits and population growth rate of the nitrogen-fixing legume *Lupinus polyphyllus*. Using seeds collected from native (North American) and invasive (Finnish) populations of the species, we conducted a greenhouse experiment in which we manipulated the soil microbiota by adding to plants either intact or autoclaved soil inoculum obtained from invasive populations. We grew the plants for two growing seasons and recorded six fitness-related traits (height early and later in life, survival, flowering probability, number of flowering shoots, inflorescence length), characterized floral volatiles, and estimated the asymptotic population growth rate (λ) using a demographic model. With the intact soil inoculum, flowering probability tended to be higher regardless of plant origin, while for early height, the effect of the inoculum treatment depended on plant origin. The other traits and floral volatile composition were not affected by soil inoculum treatment. At the population level, demographic models confirmed the positive effect of the intact soil inoculum, which yielded 130% and 30% greater λ for plants of invasive and native origins, respectively, than the autoclaved soil inoculum treatment. These results demonstrate that, at least under greenhouse conditions, soil microbiota contribute to plant population persistence and may affect species abundance. Moreover, our findings indicate that a demographic approach that considers the entire life cycle is needed to assess the net effect of soil microbiota on plant populations.

## Teaser

We investigated the effects of soil microbiota on the fitness-related traits and population growth rate of the nitrogen-fixing legume Lupinus polyphyllus using seeds collected from native (North American) and invasive (Finnish) populations of the species. Our results demonstrate that, at least under greenhouse conditions, soil microbiota contribute to plant population persistence and may affect species abundance. Importantly, our findings indicate that the net effect of soil microbiota on plant population growth rates may not be evident from individual fitness-related traits; instead, a demographic approach is needed that considers the entire life-cycle.

## Introduction

Soil microbiota can play a pivotal role in plant performance, with potential consequences for the growth and persistence of plant populations (Dawson and Schrama, 2016). Beneficial soil microbes promote plant growth through nutrient acquisition (e.g. nitrogen-fixing bacteria, mycorrhizal fungi) and can protect plants against pathogens and abiotic stress (reviewed in Hayat *et al.* 2010; Begum *et al.* 2019; Chauhan *et al.* 2023). A symbiotic partner in soil can result in considerable benefits for plant growth, particularly in nutrient-poor environments (Thrall *et al.* 2007, 2011; Hoeksema *et al.* 2010). On the other hand, pathogenic soil microbes cause diseases (Janvier *et al.* 2007) and negatively affect plant fitness (Jarosz and Davelos 1995; Mills and Bever 1998). The abundance of such antagonistic microbes in the soil is expected to accumulate over time, limiting the growth of plant populations (Dostál *et al.* 2013; Flory and Clay 2013). The nature of soil microbes (antagonists vs. mutualists) can be particularly important for invasive plant species because soil microbiota may contribute to their invasion success in new areas (Parker 2001; Simonsen *et al.* 2017; Harrison *et al.* 2018). In the introduced range, plants may encounter more or better (novel) mutualists as postulated by the enhanced mutualism hypothesis (Reinhart and Callaway 2006) or fewer antagonists as suggested by the enemy release hypothesis (Keane and Crawley 2002), resulting in growth benefits and higher plant performance compared to the native range.

In addition to direct effects, soil microbes can indirectly influence plant performance by mediating interactions between plants and other species, such as herbivores and pollinators. Soil microbiota modify plant chemical signalling through changes in the volatile organic compounds (VOCs) emitted by plants (Sharifi *et al.* 2018; Escobar-Bravo *et al.* 2023), which are used to deter herbivores or attract pollinators, as well as for chemical communication among plants (e.g. Pichersky and Gershenzon 2002; Brosset and Blande 2022). Volatile production, in turn, is modulated by phytohormones that respond differentially to antagonistic and mutualistic soil microbes (Sharifi *et al.* 2018; Sarkar and Sadhukhan 2023). For example, in the perennial *Mentha piperita*, inoculation with growth-promoting rhizobacteria enhanced the total VOC production of shoots three-fold (del Rosario Cappellari *et al.* 2017). In the annual *Brassica rapa*, plants grown under sterile conditions without microbes emitted different bouquets of floral VOCs than those inoculated with a natural floral microbiome (Helletsgruber *et al.* 2017). Because floral volatile emissions have been found to mediate pollinator choice (Dudareva *et al.* 2013; Byers *et al.* 2014; Schiestl 2015), they also have the potential to contribute to plant reproductive success.

The effects of soil microbes on plants are typically assessed by recording plant size or biomass in manipulative growth experiments that last up to a few months (Brinkman *et al.* 2010; Kardol *et al.* 2013). For perennial species, the results from short-term studies focussed on individuals’ performance early in life cannot necessarily be extrapolated to performance later in life. The effects of soil microbiota on plants may vary across different phases of the life cycle (Kardol *et al.* 2013), and thus may only be evident in longer-term studies. As an example, for three perennial grassland herbs, the presence of soil biota resulted in greater plant biomass at the seedling stage, but reduced performance at the adult stage in terms of biomass and reproduction (Dudenhöffer *et al.* 2018). Soil microbiota may also affect seedling establishment and seedling survival (e.g. Aldorfová *et al.* 2020; Sun and He 2010; Duell *et al.* 2022). Therefore, an approach that considers the entire life cycle of a given plant species may be better suited for quantifying the net effect of soil microbiota on longer-lived plants. Moreover, at the population level, only fitness-related plant traits can be expected to have an impact (Dawson and Schrama 2016) and, consequently, contribute to plant species abundances.

One useful tool for estimating the net effect of soil microbiota on plant populations is a structured demographic model, because it translates the performance of individual plants to the population level through vital rates such as survival, growth, and fecundity. Vital rates are closely linked to an individual’s fitness in addition to being crucial components of the asymptotic population growth rate (λ) which describes the finite rate of increase of the population (Caswell 2001). Importantly, changes in vital rates and λ are not interchangeable; considerable changes in the former may still have only a minor impact on λ (Ehrlén 2003; Kolb *et al.* 2010). The population-level impact of changes in vital rates depends on the sensitivity of λ to those vital rates (Caswell 2001; Ehrlén 2003; Kolb *et al.* 2010). Although this type of demographic approach has been widely used to assess the effects of a suite of environmental factors or management actions on plant populations (reviewed in Crone *et al.* 2011; Ehrlén *et al.* 2016), it has rarely been applied to studies of plant–soil microbe interactions (Ehrlén *et al.* 2016; but see Collins *et al.* 2019; David *et al.* 2019; Dostál *et al.* 2022).

Here, we investigated the effects of soil microbiota on the performance of the nitrogen-fixing perennial legume *Lupinus polyphyllus* (Lindl.) at both the individual and population levels. This species can form a symbiosis with nitrogen-fixing bacteria, rhizobia, that are acquired from soil and that are hosted in root nodules (Eckstein *et al.* 2023). Using seeds collected from native (North American) and invasive (Finnish) populations of the species, we conducted a greenhouse experiment in which we manipulated the soil microbiota by adding to each potted plant a small amount of either intact or autoclaved soil inoculum obtained from invasive populations. The use of soil microbiota from the introduced range enabled us to compare microbial effects on fitness-related traits and the population growth rate of plants in their native and introduced ranges.

We examined the effects of the soil microbiota on six fitness-related plant traits (height early and later in life, survival, flowering probability, number of flowering shoots, inflorescence length) and floral volatiles. We quantified the net effect of soil microbiota on the asymptotic population growth rate (λ) based on a demographic model (an integral projection model [IPM]). We hypothesized that plants receiving the intact soil inoculum would exhibit improved performance in terms of fitness-related traits and λ than those receiving the autoclaved soil inoculum in which microbial abundance had been reduced. In particular, we expected to observe higher performance in the intact soil inoculum treatment for plants of invasive origin because of more/better soil mutualists (growth-promoting rhizobia) or fewer soil-borne pathogens in the introduced range (Reinhart and Callaway 2006). Finally, we predicted that, as previously reported for the leaf volatiles of the study species (Kalske *et al.* 2022), the soil microbiota would modify the composition of floral volatiles, with the intact soil inoculum treatment resulting in more diverse volatile bouquets dominated by monoterpenes and sesquiterpenes (Dobson *et al.* 1996) than the autoclaved soil inoculum treatment.

## Materials and methods

### Study species

*Lupinus polyphyllus* (garden lupin, Fabaceae) is a short-lived perennial herb, 50–100 cm high, that is native to western North America and invasive at least in eastern North America, Central and South America, Europe, Australia, and New Zealand (Eckstein *et al.* 2023). Individuals typically flower in their second year at the earliest by producing one or multiple racemes of 15–60 cm that contain a large number of cross-pollinated flowers (Eckstein *et al.* 2023). Floral volatiles primarily consist of monoterpenes and sesquiterpenes (Dobson *et al.* 1996). In the study area, Finland, the species associates with rhizobia from the family Bradyrhizobiaceae (Ramula *et al.* 2023), which have also been shown to provide benefits to plants of native origin (Kalske *et al.* 2022).

### Greenhouse experiment

Due to COVID (coronavirus disease)-related travel restrictions, we used seeds collected from five native North American (USA) and five invasive Finnish (FI) populations in July 2018, from 18 to 20 haphazardly chosen maternal plants in each population. Seeds were stored in paper bags at room temperature until the experiment. The native US populations are located further south compared to the FI populations (Supplementary Table S1), but the annual mean temperatures and among-population distances are similar between the two ranges (USA 225 km, FI 238 km; Ramula and Kalske 2020). For the inoculation treatment, we collected 10 L of soil from three sites in southwestern Finland in May 2021; all of these sites were different to those from which the seeds were collected (Supplementary Table S1). At each site, we collected the soil from the rhizosphere of *L. polyphyllus* at a depth of 10 cm, with > 5 locations per site. For biosecurity reasons, only local soils were used in the experiment, which limits our comparisons to plants between native and introduced ranges. These populations have existed since at least 2010 and consist of thousands of individuals (Ramula 2014), with the mean cover of the invader being 55%–68% and grass species (Poaceae) being also abundant. We autoclaved half of the soil from each site twice at 120°C, 1 bar, for 20 min. This treatment considerably reduces the amount of viable microbes present, resulting in about 94% ± 3% (SD, standard deviation) lower microbial cover on tryptone-yeast agar plates after 10 days of incubation compared to intact soil (Ramula and Kalske 2023).

At the end of May 2021, we used four seeds from each of 18–20 maternal plants per population for the greenhouse experiment. We surface-sterilized the seeds in 0.5% commercial bleach (sodium hypochlorite) solution for 15 min and rinsed them three times with deionized water to remove epiphytic microbes. Due to asynchronous seed germination (Eckstein *et al.* 2023), we scarified the seeds by nicking the seed coat with a scalpel, sowed them on a sterilized paper towel (autoclaved at 120°C, 1 bar, 20 min) in an aluminium dish covered with plastic wrap, and kept them at room temperature and under natural light until germination. Five days after sowing, we planted seedlings in 1-L pots in a sterilized 1:1 mix of sand and potting medium (brand: Kekkilä kasvuturve, lightly fertilized with NPK and autoclaved as above). To establish the soil microbial treatments, we added 0.5 dL of the intact or autoclaved soil inoculum from one of the three sites to the surface of the sterilized growth medium and mixed it lightly to a depth of 2 cm (10 seedlings from each population per soil inoculum source). Our inoculum was roughly equivalent to 5% of the total pot volume, which is sufficient to establish a representative soil microbial community (Howard *et al.* 2017), but unlikely to alter the nutrient content of the growing substrate (Brinkman *et al.* 2010). We placed the plants in a greenhouse with ambient light and temperature, and fitted the pots (spaced ca. 10 cm apart) with bottom watering trays and a drip watering system. There were 300 plants in total (2 × ranges × 5 populations × 3 inoculum origins × 2 inoculum treatments × 5 blocks). To ensure an even distribution of treatments and populations in the greenhouse, we arranged the pots in five blocks with six plants from each population, one from each combination of inoculum origin by inoculum treatment.

From each plant we recorded height, number of leaves, and diameter at the base three weeks after potting (early in life) and twice thereafter at ca. 3-week intervals during the first growing season; none of the plants flowered in this period and this frequency was sufficient for demographic analysis. At the end of the first growing season (mid-August 2021), we destructively harvested 80 plants for a completely separate study, leaving 220 plants in the present study. The harvested plants were evenly distributed among populations, treatments, and blocks. The remaining 220 plants overwintered at + 4°C and resumed growing in March of the second year (2022). At the end of May 2022, when the plants were fully grown, we measured their height and diameter at the base, and recorded the number of flowering shoots before terminating the experiment. In all analyses, we chose to use plant height as a proxy for size.

### VOC measurements

To determine the effects of soil microbiota and plant origin on floral scent, we sampled floral VOCs from 30 plants (representing both origins and soil treatments) that flowered in May 2022 using dynamic headspace sampling. We collected samples between the hours of 9:00–12:00 on seven days over the course of three weeks, always sampling the individuals that were in full bloom. We sampled 2–9 individuals per day from both inoculation treatments to ensure that the timing of sampling did not bias our results. Samples were collected in the greenhouse under ambient light conditions and within a temperature range of 17.4–20.1°C. Each inflorescence was enclosed in a plastic oven bag (polyethylene terephthalate; 25 × 38 cm; Look® uunipussi Eskimo oy) that was tightly wrapped around its base. Activated charcoal–filtered air was then introduced to the bags, first at a rate of ca. 700 ml/min for 10 min to flush the bags, then at a rate of 225 ml/min for sample collection (pump Thomas 12v). We trapped VOCs by pulling the headspace through stainless steel tubes filled with 200 mg Tenax TA 60/80 adsorbent (Markes International Ltd) for 1 h at a flow rate of 200 ml/min with a vacuum pump (KNF). We calibrated airflows with a flowmeter every morning before trapping started (mini-Buck Calibrator, Buck) and collected one ambient control sample (an empty headspace bag) to identify potential contaminants. After the VOC sampling, we measured the length of the sampled inflorescence and counted the number of flowers.

VOC samples were stored at 4°C until analysis by gas chromatography–mass spectrometry. The trapped compounds were desorbed at 250°C for 10 min with a thermal desorption unit (TD-100; Markes International Ltd), and cryofocused at − 10°C in splitless (12 samples) or split mode (18 samples) onto an HP-5 capillary column (60 m, 250 μm × 0.25 μm; Agilent). The change from splitless to split mode was done due to an unexpectedly heavy loading of the samples. The oven temperature started at 40°C and was held for 4 min before ramping by 5°C min^−1^ to 210°C, further ramping was done at a rate of 20°C min^−1^ to a final temperature of 280°C, which was then held for 6.5 min. The carrier gas was helium with a constant flow. The transfer line temperature to the MSD was 300°C, the ionization energy was 70 eV and the full scan range of 35–430 *m/z* was used.

Sample spectra were analysed using ChemStation software. We identified compounds by comparing their mass spectra with those of pure standards (Sigma-Aldrich, Germany) and/or compounds in the NIST library (version 20). For compounds without standards, we compared their relative retention times to published Kovats retention indices to verify compound identity (Skaltsa *et al.* 2003; Adams 2017). We excluded compounds that were present in blank samples or were otherwise suspected to be contaminants, compounds for which we could not achieve reliable identification, and compounds that have not been previously reported in floral volatiles (El-Sayed 2024). Because we ran 18 of our 30 samples in split mode, we could not reliably compare peak areas across all samples and therefore only quantified presence-absence for each compound. We identified 21 monoterpenes and monoterpenoids, 23 sesquiterpenes, 9 phenylpropanoids-benzenoids, and 2 other compounds (55 compounds altogether; Supplementary Table S2). Two of our samples were too overloaded to allow even qualitative data extraction and were therefore removed, leaving us with 28 samples (intact *n* = 17, autoclaved *n* = 11, representing plants of both origins).

### Statistical analysis

We examined the effects of soil microbiota and plant origin on individuals’ height early in life in year *t* (June 2021) and on five other fitness-related traits (height later in life [log-transformed], survival, flowering probability, number of flowering shoots, inflorescence length [log-transformed]) in year *t *+ 1 (May 2022) with linear mixed models (LMM) and generalized linear mixed models (GLMM) in R software (R Core Team 2023). We used an LMM for height, number of flowering shoots, and inflorescence length (lme4::lmer) and a GLMM with a binomial distribution and logit link for survival and flowering probabilities (lme4::glmer). All models contained soil inoculum treatment (intact, autoclaved), seed origin (native USA, introduced FI), their interaction, and soil inoculum source (three sites) as fixed explanatory variables. No other interactions were considered because they were not of our interest. Population and block (physical grouping of the plant in the greenhouse) were used as random factors.

For the LMMs, we verified model assumptions visually from residual plots and transformed the response variable when necessary to improve the normality of the residuals (see above for details). For the GLMMs, we explored the residual plots for potential overdispersion using the DHARMa package (Hartig 2018) and found none (dispersion factor: 0.42–1.02). We evaluated the significance of the fixed variables with an *F* test based on the Kenward–Roger method for the LMMs (lmerTest::anova; Kuznetsova *et al.* 2017) and with a Wald chi-square test for the GLMMs (car::Anova; Fox and Weisberg 2019). We also assessed model fit based on Nakagawa’s *R*^2^, which is suited for mixed models (performance::r2_nakagawa; Lüdecke *et al.* 2021).

We tested for differences in the number of flowers on the inflorescences that were sampled for VOCs between plants treated with intact or autoclaved inoculum using a paired t-test (stats::t.test). As the autoclaved and intact treatments did not differ in inflorescence length or the number of flowers per sampled inflorescence (see Results), we examined the effect of the inoculum treatment on the composition of floral VOC emissions with a non-metric multidimensional scaling analysis using all 55 detected compounds (vegan::metaMDS; Oksanen *et al.* 2022). We used a Bray–Curtis dissimilarity matrix for binary data (vegan::vegdist) and two dimensions for the ordination (stress = 0.125). We then tested whether soil inoculum treatment affected floral VOC composition using the same dissimilarity matrix and permutational analysis of variance (9999 permutations; vegan::adonis). We did not analyse the effect of plant origin on floral VOC emissions because inflorescences were different in size between the two plant origins (see Results) and we were not able to account for the difference with the binary VOC data.

### Demographic model

To quantify the net effect of the soil microbiota on the asymptotic population growth rate (λ), we used an integral projection model. Specifically, we implemented a deterministic IPM that describes vital rates (i.e. survival, growth, fecundity) at year *t *+ 1 as a continuous function of an individual’s size in the previous year (*t*), resulting in a discretized matrix (Merow *et al.* 2014). We used log-transformed plant height from July 2021 as a continuous size variable. To compare the net effect of soil microbiota on λ between plants from the native and introduced ranges, we constructed an IPM separately for the FI and US plants in the two soil inoculum treatments (intact, autoclaved; Supplementary Appendix S1). We adopted the IPM that has been previously used for the study species (Ramula 2020), which describes population dynamics based on the following two equations:


$$\begin{array}{l} S\left(t+1\right)=s_{sb}\left(1-e\right)S\left(t\right)+\overset{U}{\underset{L}{\int }}p\left(x\right)f_{1}\left(x\right)seed\left(x\right)\left(1-e\right)n\left(x,t\right)dx \end{array}$$ (1)

$$\begin{array}{lll} n(y,t+1)=\; & e_{sb}f_{d}(y)S(t)+\overset{U}{\underset{L}{\int }}[s(x)g(y,x)\; & +p(x)f_{1}(x)seed(x)\;ef_{d}(y)]n(x,t)dx \end{array}$$ (2)

The first equation describes the total number of seeds in the seed bank at year *t *+ 1. Seeds come from two sources: seeds that survive in the seed bank from the previous year (*s*_sb_) and do not establish (*e,* the first portion of the equation), and new seeds that enter the seed bank as a result of reproduction (the second portion of the equation), in which *p*(*x*) denotes the flowering probability of individuals of size *x*, *f*_1_(*x*) is the number of flowering shoots produced of individuals of size *x*, and *seed* is the average number of seeds per flowering shoot. The second equation describes the number of plants and their sizes (*y*) present in the population at year *t *+ 1. These individuals come from two sources: establishment through germination from the seed bank, *e*_sb_ (the first portion of eq. 2), and direct establishment from seeds that never enter the seed bank (the second portion of eq. 2). In Eq. 2, *f*_d_(*y*) is the probability distribution of plant size early in life, *s*(*x*) is survival of individuals of size *x*, and *g*(*y*, *x*) is growth of individuals of size *x*. As the models were constructed based on the greenhouse experiment, in which the flowering plants did not produce seeds due to pollen limitation, we completed the life cycle by estimating seed production from field populations in July–August 2022. In each field population, we haphazardly chose 15 plants and counted the number of pods per inflorescence, as well as the number of seeds per pod from three pods per plant. We then averaged these population-specific estimates per region (Table 2), and multiplied them by the number of flowering shoots of a given plant in the greenhouse to estimate total seed production. The greenhouse data also lack information on seed survival in the seed bank and seedling establishment; we therefore used constant estimates for these parameters across soil inoculum treatments and seed origins based on previously published data from FI populations as following. Plant establishment (*e* and *e*_sb_) was set to 0.122, as in Ramula (2020), and seed survival in the seed bank (*s*_s_) was set equal to the seed viability of 0.977 estimated from 20 invasive populations based on a tetrazolium test (Ramula 2016).

**Table 2.** Estimates of vital rates (intercept + slope) and the sample sizes that were used to construct IPMs for the perennial herb *Lupinus polyphyllus* in relation to soil inoculum (intact, autoclaved) and plant origin (invasive FI, native USA). Constant estimates were used for direct plant establishment (*e* = 0.122), establishment from the seed bank (*e*_sb_ = 0.122), seed survival in the seed bank (*s*_sb_ = 0.977), and no. seeds per flowering shoot (seed) = 79 and 42 for invasive FI and native USA plants, respectively.

	Intact soil inoculum		Autoclaved soil inoculum	
Vital rate	Estimate (SE)	*n*	Estimate (SE)	*n*
Invasive (FI)				
Survival (*s*)	1.13 (5.93) + 1.16 (1.89)	220	1.13 (5.93) + 1.16 (1.89)	220
Growth (*g*), variance (σ^2^)	4.40 (0.48) − 0.16 (0.15), σ^2^ = 0.22	56	2.48 (0.46) + 0.44 (0.15), σ^2^ = 0.19	53
Flowering probability (*p*)	7.89 (6.43) − 3.09 (2.06)	56	0.27 (8.82) − 1.00 (2.81)	53
No. flowering shoots (*f*_1_)	2.67 (0.81) − 0.49 (0.28)	46	2.67 (0.81) − 0.49 (0.28)	46
Log size early in life (*f*_d_)	*μ = *2.52, σ^2^ = 0.19	55	*μ = *2.38, σ^2^ = 0.22	55
Native (USA)				
Survival (s)	1.13 (5.93) + 1.16 (1.89)	220	1.13 (5.93) + 1.16 (1.89)	220
Growth (*g*), variance (σ^2^)	3.06 (0.58) + 0.19 (0.21), σ^2^ = 0.36	55	2.91 (0.61) + 0.26 (0.21), σ^2^ = 0.30	51
Flowering probability (*p*)	−0.15 (4.99) − 0.42 (1.79)	55	−8.38 (5.36) + 2.45 (1.88)	51
No. flowering shoots (*f*_1_)	2.67 (0.81) − 0.49 (0.28)	46	2.67 (0.81) − 0.49 (0.28)	46
Log size early in life (*f*_d_)	*μ = *2.15, σ^2^ = 0.35	55	*μ = *2.12, σ^2^ = 0.26	55

**Figure 1.**
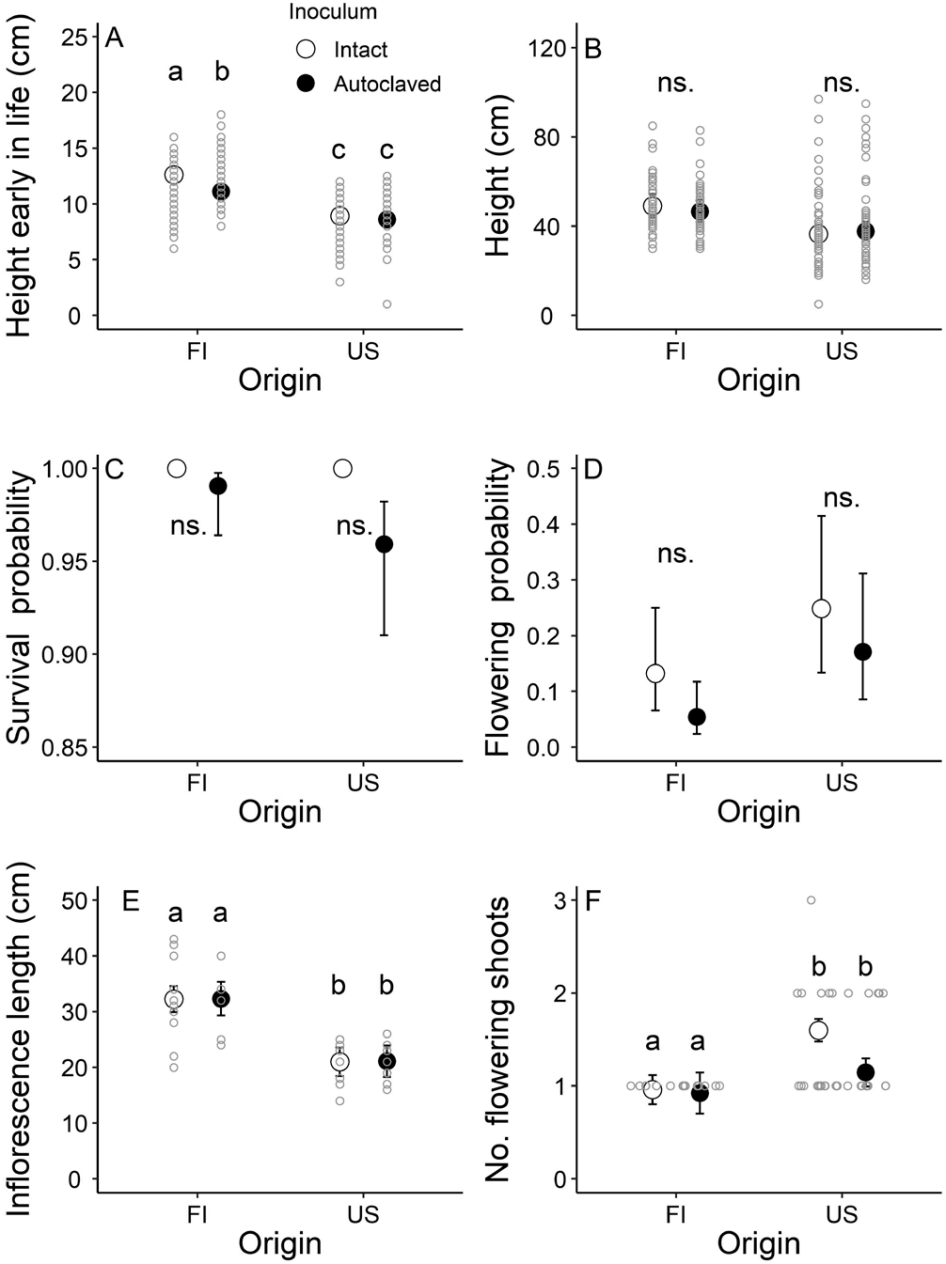
Effects of two soil inoculum treatments (intact, autoclaved) on fitness-related traits of individuals of the perennial herb *Lupinus polyphyllus* of different origins (invasive FI, native USA) grown in a greenhouse (back-transformed marginal mean ± SE with individual raw data points). Significant differences are indicated by different letters (*P *< .05, Tukey’s test or mixed model). Note that SEs are not visible for all traits because of their small size or a lack of variation in the data (for survival), and that raw data points are not shown for survival and flowering probabilities because of their original binomial scale.

We estimated treatment-specific plant traits (when possible) for the IPMs as a function of plant size from the previous year (July 2021) separately for the plants of invasive and native origins using LMMs and GLMMs with population and block as random effects. LMM was used to model plant growth and the number of flowering shoots for year *t* + 1 (late May 2022) in relation to plant size (height) from year *t* (July 2021). Survival and flowering probabilities at year *t *+ 1 were modelled in relation to plant size from year *t* with a binomial logit-link GLMM. Due to high plant survival (97.7%) and low flowering probability (Fig. 1d), we estimated survival and the number of flowering shoots from the pooled data (across the two soil inoculum treatments and two plant origins), and used constant estimates for the IPMs (see Table 2). In all these models, plant size was log-transformed and only linear relationships between plant size and the vital rates were allowed to avoid potential spurious nonlinear relationships that may arise due to the small size of the dataset. Previous work on the study of species has shown that these relationships tend to be linear (Ramula 2014).

Each IPM was discretized into a 50 × 50 matrix, in which the first class consisted of the persistent seed bank. The lower size limit in the model (L) was defined as 0.9 × the minimum observed plant size during the greenhouse study, and the upper size limit (U) was defined as 1.1 × the maximum observed plant size during the study. We explored potential eviction in the models (Williams *et al.* 2012), but found none. The asymptotic population growth rate (λ) was calculated as the leading positive eigenvalue of the discretized matrix (popbio::lambda; Stubben and Milligan 2007), and the 95th percentile confidence intervals of λ were calculated based on 1000 bootstrap replicates (Caswell 2001). The IPMs used here thus describe plant population dynamics in the greenhouse for the time interval of about 1 year (from July 2021 to late May 2022).

### Sensitivity analysis

To assess the robustness of model predictions, we conducted sensitivity analysis separately for plants of native and invasive origins based on the two soil inoculum treatments. We examined the sensitivity of λ to proportional changes in the model parameters by manually reducing each model parameter one at a time by 1% of its observed value (intercept and slope separately, Table 2) and quantifying the difference in λ.

## Results

### Fitness-related plant traits

Plant height early in life depended on the interaction between the soil inoculum treatment and plant origin (Table 1). Although the invasive FI plants were generally taller than the native US plants, the intact soil inoculum treatment increased the size of the former by 13.6% compared to the autoclaved inoculum treatment. Instead, soil treatment had no effect on plants of native origin (Fig. 1a). Moreover, plant height early in life depended on soil inoculum source (Table 1), with inoculum collected from site 3 being particularly beneficial (Supplementary Table S3). Plant height and survival at the end of the experiment (May 2022) did not differ between soil inoculum treatments, plant origins, or soil inoculum sources (Table 1; Fig. 1b and c). Flowering probability did not differ between plant origins, but tended to be higher for the plants grown with the intact soil inoculum than for those grown with the autoclaved soil inoculum irrespective of plant origin (a result that did not, however, quite meet the threshold of statistical significance; Table 1; Fig. 1d). Number of flowering shoots and inflorescence length were not affected by soil inoculum treatment or soil inoculum source, but differed between plant origins (Table 2), with the invasive FI plants producing 31.5% fewer flowering shoots and 58% longer inflorescences than the native US plants (Fig. 1e and f).

**Table 1.** Results from LMM and GLMM mixed models of the effects of soil inoculum, plant origin, and soil inoculum source on six fitness-related traits of the perennial herb *Lupinus polyphyllus* in year *t* or *t* + 1. Population and block were included as random factors for all models. df and ddf denote the degrees of freedom in the numerator and denominator for the LMM, respectively (for the GLMM df is 1). Significant effects (*P *< .05) are in bold and a marginally significant effect is indicated with an asterisk (*). *R*^2^ refers to the explanatory power of the fixed factors in the model.

	Early height (*t*)		log Height (*t* + 1)		Survival (*t* + 1)		Flowering prob. (*t* + 1)		No. fl. shoots (*t* + 1)		log Inflorescence length (*t* + 1)	
Explanatory variable	*F*_df, ddf_	*P*	*F*_df, ddf_	*P*	χ^2^	*P*	χ ^2^	*P*	*F*_*df, ddf*_	*P*	*F*_df, ddf_	*P*
Soil inoculum (intact, autoclaved)	**11.768**_**1,202**_	**0.001**	0.074_1,198_	0.785	0.000	1.000	3.048	0.081^*^	2.379_1,39_	0.131	0.000_1,20_	.975
Origin (FI, US)	**68.473**_**1,8**_	**<0.001**	3.254_1,8_	0.108	0.892	.345	1.301	0.254	**6.013**_**1,7**_	**0.047**	**18.699**_**1,4**_	**.013**
Inoculum source (3 sites)	**6.122**_**2,205**_	**0.003**	0.048_1,202_	0.953	0.444	.801	0.475	0.789	1.046_2,37_	0.361	0.966_3,23_	.385
Soil inoculum × Origin	**4.801**_**1,203**_	**0.030**	1.260_1,199_	0.263	0.000	1.000	0.419	0.517	1.800_1,7_	0.187	0.000_1,23_	.999
*R* ^2^	0.403		0.120		0.959		.074		0.266		0.556	

### Population growth rates and sensitivity analysis

**Figure 2.**
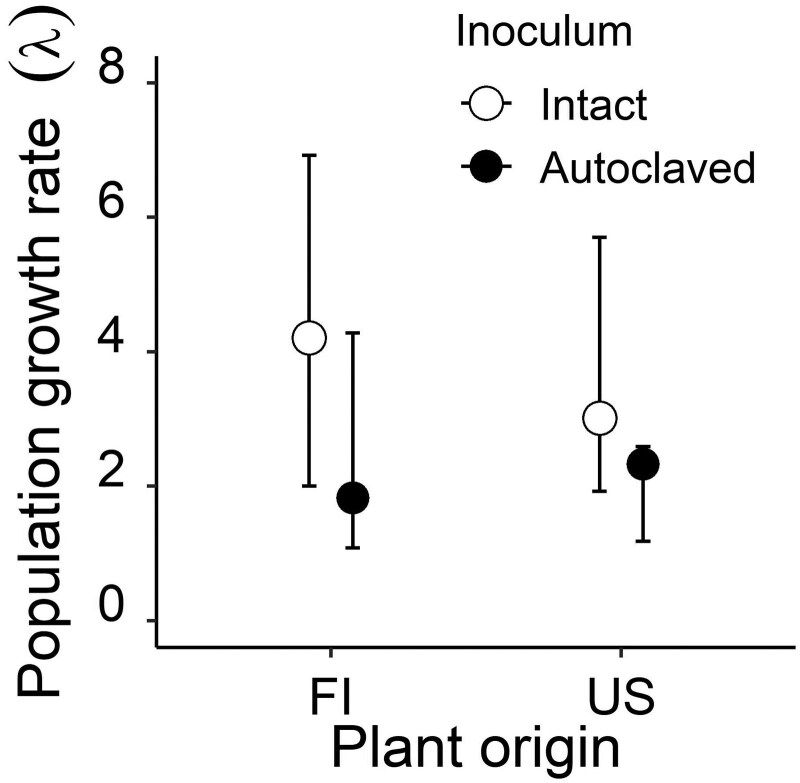
Asymptotic population growth rates (λ) estimated from two soil inoculum treatments for the perennial herb *Lupinus polyphyllus* grown in a greenhouse from seeds obtained from two different origins (invasive FI, native USA). Bars show 95th percentile confidence intervals based on 1000 bootstrap replicates.

All population growth rates (λ) estimated were high (range: 1.83–4.21), meaning that the populations were predicted to roughly double or increase their size fourfold during the study period. This growth rate was affected by the soil inoculum treatment, with the intact soil inoculum resulting in higher λ than the autoclaved soil inoculum for plants of both origins (Fig. 2). However, the positive effect of the intact soil inoculum on λ was more pronounced for plants of invasive origin than for those of native origin (130% vs. 30% increase in λ, respectively; Fig. 2).

**Figure 3.**
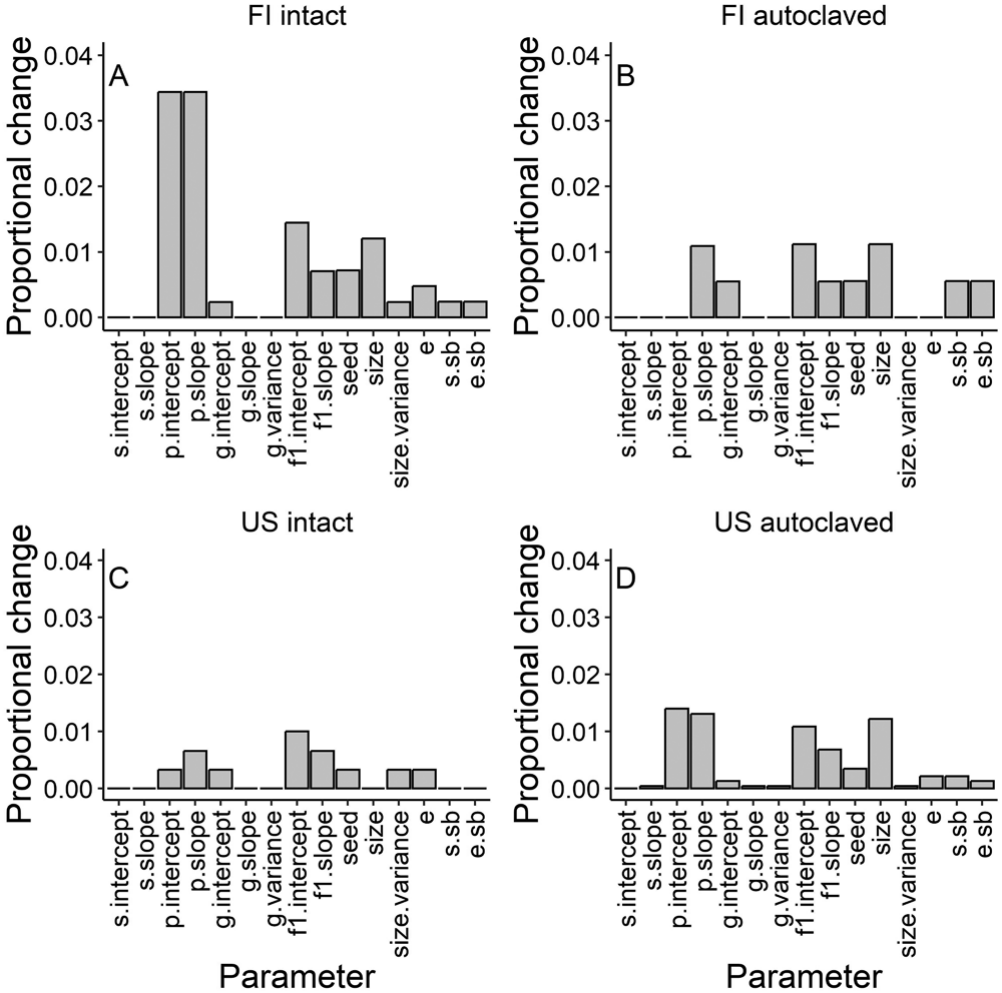
Sensitivity of asymptotic population growth rates (λ) to relative changes in the model parameters for the perennial herb *Lupinus polyphyllus* grown in two soil inoculum treatments. Plants originated from a) invasive FI and b) native US populations. Sensitivities were calculated as the proportional difference compared to the observed λ and are shown as absolute values. Abbreviations: s = survival, p = flowering probability, g = growth, f1 = number of flowering shoots, seed = seeds per flowering shoot, e = direct plant establishment, s.sb = survival in the seedbank, e.sb = establishment from the seedbank (see Table 2).

Sensitivity analysis revealed that the population growth rates of FI and US plants in both soil inoculum treatments were most sensitive to proportional changes in flowering probability and the number of flowering shoots, while proportional changes in survival had a minor effect (Fig. 3). Moreover, the population growth rates of the FI plants in both soil inoculum treatments and US plants treated with the autoclaved soil inoculum were sensitive to proportional changes in plant size early in life (Fig. 3).

### Floral volatiles

The inflorescences sampled for floral VOCs did not differ in terms of the number of flowers between the two soil inoculum treatments (*t* = 0.81, df = 26, *P* = .426, mean ± SE (standard error) = 68.9 ± SE 5.8 for intact and 62.1 ± SE 5.4 for autoclaved). Moreover, soil inoculum treatment did not appear to affect the composition of floral VOC emissions (pseudo-*F* = 1.80, *P* = .341, Supplementary Fig. S1). Compounds that were present in most samples included the monoterpenes: myrcene, limonene, β-ocimene, linalool and the sesquiterpenes: (*E*)-caryophyllene and (*Z*)-β-farnesene (Supplementary Table S3).

## Discussion

Of the six fitness-related traits we measured on the herbaceous legume *Lupinus polyphyllus* grown under greenhouse conditions, soil microbiota affected only height early in life, with plants of invasive FI origin benefitting from the intact soil inoculum treatment more than plants of native US origin. Moreover, plants grown with the intact soil inoculum tended to have a higher flowering probability than those grown with the autoclaved soil inoculum in which microbial abundance was reduced, but this trend was not statistically significant. The asymptotic population growth rates (λ) were greater for plants treated with the intact soil inoculum compared to the autoclaved soil inoculum, confirming the positive net effect of soil microbiota on *L. polyphyllus* at the population level. As predicted, the positive net effect of the intact soil inoculum treatment on λ was greater for plants of invasive origin (130%) than for plants of native origin (30%). However, contrary to our expectations, we detected no effect of soil inoculum on floral VOC composition.

At the individual level, plants of invasive FI origin appeared to benefit from the intact soil inoculum more than plants of native US origin in terms of height early in life (13.6% vs. no increase in height, respectively). Nevertheless, most plant traits (height later in life, survival, number of flowering shoots, inflorescence length), were unaffected by soil microbiota. These results are quite opposite to our previous findings in the same study system, in which we observed, under greenhouse conditions, greater benefits of soil microbiota to plants of native origin in terms of biomass and number of leaves (Kalske *et al.* 2022). In the present study, the greater benefit of soil microbiota to invasive FI plants early in life is, however, in line with the enhanced mutualism hypothesis (Reinhart and Callaway 2006), suggesting that plants in the introduced range benefit from local mutualistic partners. Alternatively, the release from soil-borne pathogens (Keane and Crawley 2002) could explain the observed difference. The greater benefit of soil microbiota to invasive FI plants could also be due to local adaptation, because all soil microbiota were obtained from invasive populations and were putatively novel to the native US plants. Regardless of the exact mechanism for the differences in fitness-related traits between plants of different origins, our results suggest that the positive effects of soil microbiota from the introduced range outweighed the potential negative effects of pathogens on the study species. These positive effects were probably due to mutualistic partners in the invaded soils, presumably nitrogen-fixing bacteria belonging to the family Bradyrhizobiaceae (Ramula *et al.* 2023). A similar positive effect of soil microbiota has also been reported for FI plants of *L. polyphyllus* that were grown in non-native soil in the USA (Sirivat *et al.* 2023).

The negligible effects of soil microbiota on fitness-related traits at the adult stage observed here might be due to the low amount of soil inoculum used in the experiment (about 5% of the total pot volume). In the inoculation-based study approach, soil microbial abundance is diluted compared to that in natural conditions to avoid creating changes in soil nutrient content (Brinkman *et al.* 2010). In our case, though, the explanation of low microbial abundance seems unlikely because the density of the soil microbiota usually increases over time and we quantified most of the plant traits at the end of the experiment, nearly a year after inoculation. Furthermore, the fact that we did detect a significant soil inoculum effect 2 months after inoculation (on early plant size) confirms that the effects of soil microbes on plant traits were already measurable early in life when microbial densities were presumably lower. Alternatively, the negligible effects of the soil microbiota on plant traits at the adult stage could be due to greater-than-expected similarities between the two soil inoculum treatments, potentially as a result of cross-contamination during our 12-month greenhouse experiment. To evaluate this hypothesis, we conducted molecular analyses on soil samples collected from the pots four months after applying the inocula (September 2021) and observed a clear difference (clustering patterns in an ordination) between the bacterial communities in the intact and autoclaved inoculum treatments (Kalske A. *et al.* unpublished data). Unfortunately, it is not known whether this difference in the soil bacterial communities persisted until the end of the experiment and which bacterial families or genera interacted with the plants.

Although the demographic models confirmed the positive net effect of soil microbiota on λ, they also suggested a much greater positive impact of soil microbes on plants of invasive origin than on plants of native origin (i.e. a different outcome from that based on most fitness-related traits). Such a mismatch between analyses based on demographic models and those based on individual fitness traits has been reported previously for perennial herbs (Ehrlén 2003; Kolb *et al.* 2010; Ramula 2014), and is primarily due to the fact that λ is differentially sensitive to different individual fitness-related traits (Ehrlén 2003). In the present study, λ tended to be most sensitive to changes in flowering probability, number of flowering shoots, and size early in life, with only the last trait being differentially affected by soil inoculum treatment between plant origins. Such high sensitivity of λ to fecundity is typical for rapidly growing plant populations (Sillvertown *et al.* 1993; de Kroon *et al.* 2000; Ramula *et al.* 2008; Ramula 2017).

Our λ estimates come with some caveats. First, we assumed constant seed survival in the seed bank and constant plant establishment across the two soil inoculum treatments and both plant origins, which may have led to underestimates of the effect of soil microbiota on λ. Plant establishment here includes seed germination and early survival, which can be critical components in a plant’s life cycle (e.g. Picó *et al.* 2003; Villellas *et al.* 2013). For legumes that associate with mutualistic rhizobia, like our study species, a lack of suitable partners can retard population establishment (Simonsen *et al.* 2017; Harrison *et al.* 2018), with seedling establishment being the fundamental step in the invasion process. As all soil microbiota came from invasive populations and were thus putatively novel for the plants of native origin, it is possible that our intact soil inoculum treatment differentially affected plant establishment depending on plant origin (e.g. poorer establishment for plants of native origin). However, sensitivity analysis conducted for the demographic models based on the FI and US plants grown with the two soil inocula revealed that their population growth rates were not particularly sensitive to changes in seed survival in the seed bank or plant establishment (Fig. 3), suggesting that our results are robust in this regard. Second, high λ estimates observed under greenhouse conditions without competitors may not be directly translatable into the field, where both intraspecific and interspecific competitors may limit population growth. Despite these caveats, we detected a positive effect of soil microbiota on λ under greenhouse conditions, which is probably due to the fact that our study species is known to associate with mutualistic rhizobia. This result is in line with previous findings from other plant species of the sometimes considerable effects of soil microbiota on plant populations. For example, for the endangered herb *Hypericum cumulicola*, soil microbes were reported to increase λ in low-nutrient environments in a greenhouse (David *et al.* 2019), and for two annual prairie herbs, arbuscular mycorrhizal fungi increased plant survival and reproduction in a common garden (Reynolds *et al.* 2020). These findings collectively indicate that soil microbiota should be explicitly considered as a potential mechanism contributing to plant species abundance and invasion success.

Here, soil microbiota had no effect on the identity of 55 floral volatiles detected in the plants grown in the intact and autoclaved soil inoculum treatments, but volatiles that are typical to bumblebee-pollinated flowers (myrcene, limonene, β-ocimene; Schiestl 2015) were present in nearly all samples. Moreover, the sesquiterpene (*E*)-β-caryophyllene, which protects against bacterial pathogens (Huang *et al.* 2012), occurred in all samples. This result stands in contrast to our previous findings on the leaf volatiles of this study species, which were observed to be affected by soil microbes (Kalske *et al.* 2022). The negligible effect of the soil microbiota on floral VOCs in the present study could be due to the fact that we used qualitative (presence-absence) rather than quantitative data on chemical compounds. Therefore, we cannot rule out the possibility that the soil microbiota might have affected the emission rates of the compounds observed in both treatments. It is also possible that floral VOCs might be primarily modified by floral microbiota rather than soil microbiota. For example, in the annual herb *Brassica rapa*, the inoculation of buds and leaves with floral bacteria affected floral scent, resulting in distinct VOC profiles between inoculated and uninoculated plants (Helletsgruber *et al.* 2017).

Overall, we demonstrate here that, for *L. polyphyllus* grown under greenhouse conditions without competitors, soil microbiota contributed to plant population persistence. Importantly, our findings indicate that the net effect of soil microbiota on plant population growth rates may not be evident from individual fitness-related traits; instead, a demographic approach is needed that considers the entire life cycle. We believe that the combination of manipulative experiments of soil microbiota with demographic modelling provides a valuable tool for better understanding and quantifying the impact of the soil microbiome on plant populations. Such population-level assessments can be particularly useful for predictions of plant species abundances and distributions.

## Supplementary Material

plaf012_suppl_Supplementary_Materials

## Data Availability

The data underlying this article are available in its online supplementary material and on Zenodo https://doi.org/10.5281/zenodo.14673130. Conflict of interest: None declared.
