# Supplementary material for: Soil microbiota enhance the population growth rate of a nitrogen-fixing herbaceous legume
Source: AoB Plants. 2025 Mar 16;17(4):plaf012. doi: 10.1093/aobpla/plaf012 (PMC12230798; doi:10.1093/aobpla/plaf012)
Supplement: plaf012_suppl_Supplementary_Materials [file plaf012_suppl_supplementary_materials.pdf]

**Supporting Information for**

# Soil microbiota enhance the population growth rate of a nitrogen-fixing herbaceous legume

Satu Ramula, James D Blande, Aino Kalske

*E-mail: satu.ramula@utu.fi

**Table S1.** Locations of the study populations for seeds (US, FI) and soil inocula (S).

| Population | Country/region | Lat, Long | Seeds per flowering shoot |
|---|---|---|---|
| **US-1** | US/CA-Stanislaus | 38.18, -120.04 | 52.4 |
| **US-2** | US/CA-Eldorado | 38.69, -120.02 | 21.3 |
| **US-3** | US/CA-Tahoe | 39.35, -120.35 | 61.3 |
| **US-4** | US/CA-Sagehen Creek | 39.43, -120.24 | 44.8 |
| **US-5** | US/OR-Winema | 42.46, -122.40 | 29.3 |
| **FI-1** | FI/Turku | 60.36, 22.27 | 94.8 |
| **FI-2** | FI/Turku | 60.41, 22.74 | 18.1 |
| **FI-3** | FI/Jämsä | 61.85, 25.17 | 79.3 |
| **FI-4** | FI/Kuopio | 62.66, 27.34 | 86.5 |
| **FI-5** | FI/Kuopio | 63.12, 27.66 | 118.0 |
| Soil site | Country/region | Lat, Long | |
| **S-1** | FI/Raisio | 60.49, 22.17 | NA |
| **S-2** | FI/Turku | 60.51, 22.30 | NA |
| **S-3** | FI/Kaarina | 60.48, 22.19 | NA |

**Table S2.** Volatile compounds collected from headspace of whole inflorescences of the perennial herb *Lupinus polyphyllus*. Compounds present in most samples are in bold.

| No. | Name | Class[a] | Formula | Validation[b] | RT | (intact+autoclaved) n |
|---|---|---|---|---|---|---|
| 1 | α-Thujene | MT | $C_{10}H_{16}$ | RT, MS | 12.89 | (9+4) 13 |
| 2 | α-Pinene | MT | $C_{10}H_{16}$ | ST, RT, MS | 13.12 | (13+9) 22 |
| 3 | Benzaldehyde | PB | $C_7H_6O$ | RT, MS | 13.91 | (12+7) 19 |
| 4 | Sabinene | MT | $C_{10}H_{16}$ | RT, MS | 14.32 | (12+5) 17 |
| 5 | β-Pinene | MT | $C_{10}H_{16}$ | ST, RT, MS | 14.51 | (5+3) 8 |
| **6** | **Myrcene** | **MT** | $\mathbf{C_{10}H_{16}}$ | **ST, RT, MS** | **14.80** | **(16+10) 26** |
| 7 | α-Phellandrene | MT | $C_{10}H_{16}$ | ST, RT, MS | 15.33 | (5+1) 6 |
| 8 | α-Terpinene | MT | $C_{10}H_{16}$ | RT, MS | 15.68 | (9+5) 14 |
| 9 | Cymene | MT | $C_{10}H_{14}$ | ST, RT, MS | 15.92 | (3+5) 8 |
| **10** | **Limonene** | **MT** | $\mathbf{C_{10}H_{16}}$ | **ST, RT, MS** | **16.12** | **(16+10) 26** |
| 11 | 1,8-Cineole | MT | $C_{10}H_{18}O$ | ST, RT, MS | 16.23 | (1+1) 2 |
| **12** | **β-Ocimene** | **MT** | $\mathbf{C_{10}H_{16}}$ | **RT, MS** | **16.59** | **(17+10) 27** |
| 13 | γ-Terpinene | MT | $C_{10}H_{16}$ | ST, RT, MS | 17.04 | (12+6) 18 |
| 14 | Acetophenone | PB | $C_8H_8O$ | RT, MS | 17.27 | (9+6) 15 |
| 15 | (*Z*)-Sabinene hydrate | MT | $C_{10}H_{18}O$ | RT, MS | 17.44 | (2+2) 4 |
| 16 | Terpinolene | MT | $C_{10}H_{16}$ | ST, RT, MS | 17.99 | (14+7) 21 |
| 17 | Methyl benzoate | PB | $C_8H_8O_2$ | RT, MS | 18.17 | (4+2) 6 |
| **18** | **Linalool** | **MT** | $\mathbf{C_{10}H_{18}O}$ | **ST, RT, MS** | **18.22** | **(16+10) 26** |
| 19 | *allo*-Ocimene | MT | $C_{10}H_{16}$ | RT, MS | 19.05 | (13+7) 20 |
| 20 | 1,3,8-ρ-Menthatriene | MT | $C_{10}H_{14}$ | RT, MS | 19.13 | (13+8) 21 |
| 21 | Veratrole | PB | $C_8H_{10}O_2$ | RT, MS | 19.48 | (0+5) 5 |
| 22 | Terpinen-4-ol | MT | $C_{10}H_{18}O$ | ST, RT, MS | 20.50 | (10+4) 14 |
| 23 | Methyl salicylate | PB | $C_8H_8O_3$ | ST, RT, MS | 20.91 | (6+6) 12 |
| 24 | α-terpineol | MT | $C_{10}H_{18}O$ | ST, RT, MS | 20.98 | (4+1) 5 |

| | | | | | | |
|---|---|---|---|---|---|---|
| 25 | 2-Aminobenzaldehyde | PB | $C_7H_7NO$ | MS | 21.38 | (11+6) 17 |
| 26 | Geraniol | MT | $C_{10}H_{18}O$ | RT, MS | 22.05 | (3+1) 4 |
| 27 | Methyl citronellate | MT | $C_{11}H_{20}O_2$ | RT, MS | 22.14 | (2+0) 2 |
| 28 | Nonanoic acid | O | $C_9H_{18}O_2$ | RT, MS | 22.22 | (5+5) 10 |
| 29 | Indole | O | $C_8H_7N$ | RT, MS | 23.02 | (5+5) 10 |
| 30 | Methyl o-anisate | PB | $C_9H_{10}O_3$ | RT, MS | 23.73 | (3+4) 7 |
| 31 | Methyl anthranilate | PB | $C_8H_9NO_2$ | RT, MS | 23.91 | (10+5) 15 |
| 32 | α-Cubebene | ST | $C_{15}H_{24}$ | RT, MS | 24.07 | (12+5) 17 |
| 33 | α-Ylangene | ST | $C_{15}H_{24}$ | RT, MS | 24.50 | (2+0) 2 |
| 34 | α-Copaene | ST | $C_{15}H_{24}$ | RT, MS | 24.59 | (15+7) 22 |
| 35 | β-Cubebene | ST | $C_{15}H_{24}$ | RT, MS | 24.78 | (10+3) 13 |
| 36 | β-Elemene | ST | $C_{15}H_{24}$ | ST, RT, MS | 24.81 | (5+6) 11 |
| 37 | Methyl N-methylanthranilate | PB | $C_8H_9NO_2$ | RT, MS | 25.05 | (5+2) 7 |
| 38 | α-Santalene | ST | $C_{15}H_{24}$ | RT, MS | 25.22 | (4+3) 7 |
| **39** | **(*E*)-Caryophyllene** | **ST** | $\mathbf{C_{15}H_{24}}$ | **ST, RT, MS** | **25.36** | **(17+10) 27** |
| 40 | *(E)*-α-bergamotene | ST | $C_{15}H_{24}$ | RT, MS | 25.44 | (13+7) 20 |
| 41 | α-Guaiene | ST | $C_{15}H_{24}$ | RT, MS | 25.54 | (6+4) 10 |
| **42** | **(*Z*)-β-Farnesene** | **ST** | $\mathbf{C_{15}H_{24}}$ | **ST, RT, MS** | **25.67** | **(17+10) 27** |
| 43 | α-Humulene | ST | $C_{15}H_{24}$ | ST, RT, MS | 25.89 | (14+9) 23 |
| 44 | *allo*-Aromadendrene | ST | $C_{15}H_{24}$ | RT, MS | 25.99 | (14+6) 20 |
| 45 | α-Curcumene | ST | $C_{15}H_{22}$ | RT, MS | 26.13 | (12+7) 19 |
| 46 | γ-Muurolene | ST | $C_{15}H_{24}$ | RT, MS | 26.14 | (6+2) 8 |
| 47 | γ-Gurjunene | ST | $C_{15}H_{24}$ | RT, MS | 26.23 | (4+3) 7 |
| 48 | Germacrene D | ST | $C_{15}H_{24}$ | RT, MS | 26.26 | (15+8) 23 |
| 49 | α-Farnesene | ST | $C_{15}H_{24}$ | RT, MS | 26.37 | (15+9) 24 |
| 50 | β-Bisabolene | ST | $C_{15}H_{24}$ | RT, MS | 26.47 | (8+3) 11 |
| 51 | β-Curcumene | ST | $C_{15}H_{24}$ | RT, MS | 26.51 | (5+3) 8 |
| 52 | β-Sesquiphellandrene | ST | $C_{15}H_{24}$ | RT, MS | 26.70 | (2+1) 3 |

| | | | | | | |
|---|---|---|---|---|---|---|
| 53 | δ-Cadinene | ST | $C_{15}H_{24}$ | RT, MS | 26.76 | (14+6) 20 |
| 54 | Selina-3,7(11)-diene | ST | $C_{15}H_{24}$ | RT, MS | 27.13 | (4+3) 7 |
| 55 | Caryophyllene oxide | ST | $C_{15}H_{24}$ | RT, MS | 27.29 | (7+5) 12 |

a: MT= monoterpenes/monoterpenoids, PB= phenylpropanoids and benzenoids, O=: other, ST= sesquiterpenes
b: ST= standard, RT= retention time, MS= NIST library match with mass spectra, >90% confidence

**Table S3.** Parameter estimates from linear (LMM) and generalized linear (GLMM) mixed models for the effects of soil inoculum treatments, seed origin, and soil inoculum source on the fitness-related traits of the perennial herb *Lupinus polyphyllus*.

| **Response variable** | **Parameter** | **Coefficient** | **SE** |
|---|---|---|---|
| Fixed factors | | | |
| Early height | Intercept (FI, intact, site1) | 12.069 | 0.437 |
| | Inoculum_autoclaved | -1.529 | 0.385 |
| | Origin_US | -3.704 | 0.464 |
| | Source_site2 | 0.520 | 0.338 |
| | Source_site3 | 1.165 | 0.333 |
| | Inoculum_autoclaved × Origin_US | 1.194 | 0.544 |
| Fixed factors | | | |
| Height | Intercept (FI, intact, site1) | 3.886 | 0.107 |
| | Inoculum_autoclaved | -0.054 | 0.054 |
| | Origin_US | -0.298 | 0.146 |
| | Source_site2 | 0.013 | 0.047 |
| | Source_site3 | 0.013 | 0.047 |
| | Inoculum_autoclaved × Origin_US | 0.086 | 0.077 |
| Fixed factors | | | |
| Survival | Intercept (FI, intact, site1) | 21.988 | 5238.474 |
| | Inoculum_autoclaved | -16.775 | 5238.474 |
| | Origin_US | -0.171 | 6731.464 |
| | Source_site2 | -0.888 | 1.399 |
| | Source_site3 | -0.793 | 1.399 |
| | Inoculum_autoclaved × Origin_US | -1.324 | 6731.464 |
| Fixed factors | | | |
| Flowering prob. | Intercept (FI, intact, site1) | -1.732 | 0.824 |

| | | | |
|---|---|---|---|
| | Inoculum_autoclaved | -0.988 | 0.609 |
| | Origin_US | 0.773 | 0.912 |
| | Source_site2 | -0.327 | 0.477 |
| | Source_site3 | -0.117 | 0.471 |
| | Inoculum_autoclaved × Origin_US | 0.515 | 0.795 |
| Fixed factors | | | |
| No. flowering shoots | Intercept (FI, intact, site1) | 0.949 | 0.170 |
| | Inoculum_autoclaved | -0.038 | 0.242 |
| | Origin_US | 0.639 | 0.182 |
| | Source_site2 | -0.113 | 0.165 |
| | Source_site3 | 0.148 | 0.160 |
| | Inoculum_autoclaved × Origin_US | -0.415 | 0.289 |
| Fixed factors | | | |
| Inflorescence length | Intercept (FI, intact, site1) | 32.419 | 1.977 |
| | Inoculum_autoclaved | 0.397 | 3.110 |
| | Origin_US | -12.426 | 2.843 |
| | Source_site2 | NA | |
| | Source_site3 | NA | |
| | Inoculum_autoclaved × Origin_US | 0.767 | 4.406 |

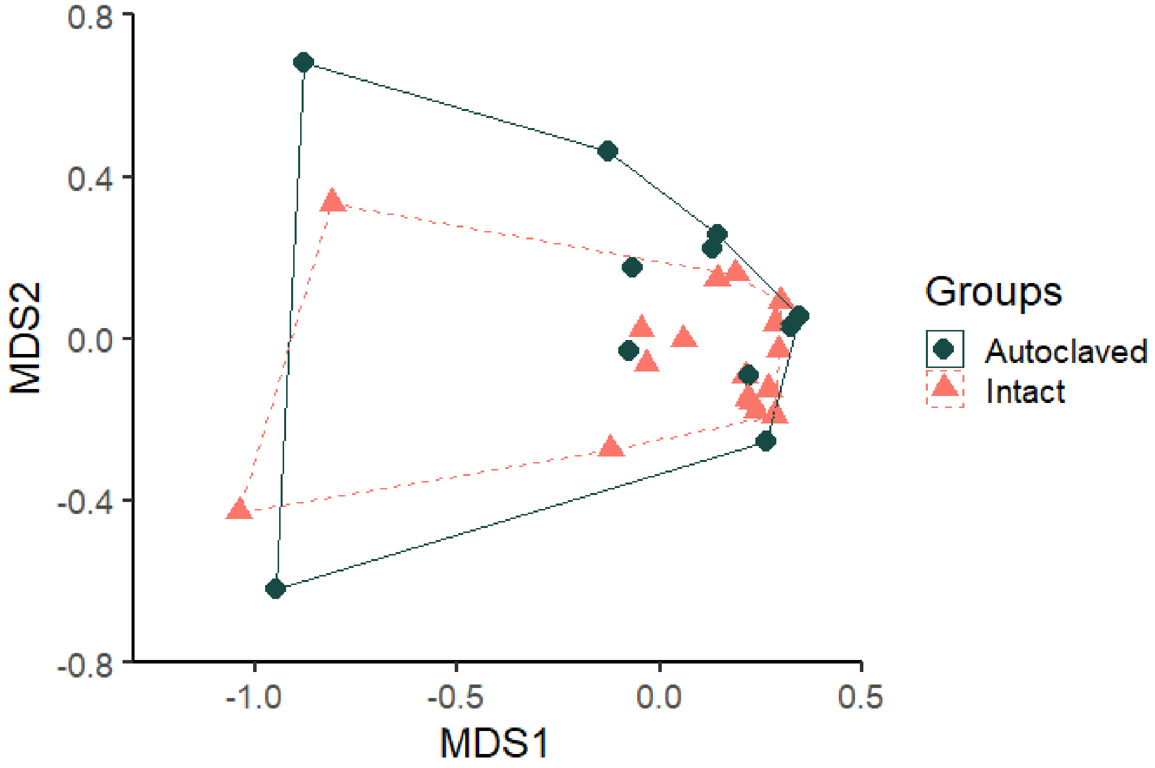


**Figure S1.** Non-metric multidimensional scaling (NMDS) ordination based on presence-absence of 55 floral volatiles sampled from the perennial herb *Lupinus polyphyllus* grown in the intact (n=17 plants) and autoclaved (n=11 plants) soil inoculum treatments. Each symbol represents an individual plant and its VOC profile.

**Appendix S1.** An R code to construct an integral projection model for the perennial herb *Lupinus polyphyllus*.

```
#Code for an IPM (without bootstrap) by Satu Ramula 26 Aug 2024 to
#estimate population growth rate for a given soil inoculum treatment
############################################################
# Install packages
library(readxl)  #read in data from Excel
library(lme4)    #for mixed models
library(popbio)  #calculate population growth rate

# Read in "Lupinus.data"
Data_IPM <- read_excel("E://Lupinus.data.xlsx")

Size.t<-Data_IPM$height.july21  #plant size at year t
Size.t1<-Data_IPM$height.may22  #plant size at year t+1
Flowering.t1<-with(Data_IPM, ifelse(fl.shoots.may22 >0, 1, 0))

#add size variables and flowering probability to the data
Data_IPM<-data.frame(Data_IPM, Size.t, Size.t1, Flowering.t1)

#======================================================
#      Models for vital rates
#======================================================

popmatrix<-matrix(nrow=50, ncol=50)   #create an empty 50 by 50 matrix

# Subset the data to construct an IPM separately for each country (FI, US)
#and treatment (autoclaved, intact)
popdata<-subset(Data_IPM, Data_IPM$treatment=="FII")   #FI intact
#popdata<-subset(Data_IPM, Data_IPM$treatment=="FIA")  #FI autoclaved
#popdata<-subset(Data_IPM, Data_IPM$treatment=="USI")  #US intact
#popdata<-subset(Data_IPM, Data_IPM$treatment=="USA")  #US autoclaved

e<-0.122            #direct plant establishment
ssb<- 0.977         #seed survival in the seed bank
esb<-0.122          #establishment from the seed bank

#########################
#Fit statistical models for flowering probability, survival, growth, and
#no. flowering shoots.
#===========================
#      Flowering probability
#==========================

f1<-glmer(as.factor(Flowering.t1)~log(Size.t)+(1|pop)+(1|block),
data=popdata, family=binomial(link="logit"), na.action=na.omit)

##save the intercept and slope, plot data
p.inter<-f1$coefficients[1]
p.slope<-f1$coefficients[2]
plot(log(popdata$Size.t), popdata$Flowering.t1)

#===========================
#      Survival
#===========================

#Use all data (Data_IPM) to estimate survival because of low mortality.
s1<-glmer(as.factor(survived.may22)~log(Size.t)+(1|pop)+(1|block),
data=Data_IPM, family=binomial(link="logit"), na.action=na.omit)
```

```
##save the intercept and slope, plot data
s.inter<-s1$coefficients[1]
s.slope<-s1$coefficients[2]
plot(log(popdata$Size.t), popdata$survived.may22)

#====================
#      Growth
#====================

g1<-lmer(log(Size.t1)~log(Size.t) +(1|pop)+(1|block), data=popdata,
na.action=na.omit)

#save the intercept, slope, and residual variance, plot data
g.inter<-g1$coefficients[1]
g.slope<-g1$coefficients[2]
v.growth<-sd(resid(g1))
plot(log(popdata$Size.t), log(popdata$Size.t1))

#==========================
#     No. flowering shoots based on plants that flowered in 2022
#==========================

#Use all flowering plants (Data_IPM)
fecdata<-subset(Data_IPM, Data_IPM$Flowering.t1==1)  #subset the data to
include all flowering plants across plant origins and treatments

fec<-lmer(shoots22~log(Size.t) +(1|pop)+(1|block),data=fecdata,
na.action=na.omit)

##save the intercept and slope, plot data
f1.inter<-fec$coefficients[1]
f1.slope<-fec$coefficients[2]
plot(log(fecdata$Size.t), fecdata$shoots22))

#=====================================================
#     IPM based on the statistical models above
#=====================================================

#meshpoints (90-110% of the observed plant size)
minsize<-min(c(log(popdata$Size.t), log(popdata$Size.t1)), na.rm = TRUE) *
0.9    #min observed size
maxsize<-max(c(log(popdata$Size.t), log(popdata$Size.t1)), na.rm = TRUE) *
1.1    #max observed size

#Treatment-specific estimates of seedling size (mean and SD) calculated
#from log-transformed greenhouse data
mean.sdl<-2.522  #mean for FI intact
var.sdl<-0.19    #SD for FI intact
#mean.sdl<-2.384 #mean for FI autoclaved
#var.sdl<-0.223  #SD for FI autoclaved
#mean.sdl<-2.146 #mean for US intact
#var.sdl<-0.354  #SD for US intact
#mean.sdl<-2.120 #mean for US autoclaved
#var.sdl<-0.261  #SD for US autoclaved

#region-specific seed number per flowering shoot estimated from the field
seedn<-79         #for FI plants
#seedn<-42        #for Us plants

n.big.matrix<-49  #size of the big matrix without 1. column (seed bank)
```

```
# All model parameters
s.inter             #intercept for survival
s.slope             #slope for survival
p.inter             #flowering intercept
p.slope             #flowering slope
g.inter             #intercept for growth
g.slope             #slope for growth
v.growth            #variance in growth
f1.inter            #intercept for no. flowering shoots
f1.slope            #slope for no. flowering shoots
mean.sdl            #mean seedling size
var.sdl             #variance in seedling size
seedn               #number of seeds per flowering shoot

################################################################
# Compute the kernel component functions from the fitted models
#################################################################

#survival function
sx<-function(x) {
  u<-(s.inter+s.slope*x)
  return(exp(u)/(1+exp(u)))   #survival is in logit-scale, back-transform
to restrict the function between 0 and 1
}

#function for flowering probability
fx<-function(x) {
  u<-(p.inter+p.slope*x)
  return(exp(u)/(1+exp(u)))   #flowering prob. is in logit-scale, back-
transform to restrict the function between 0 and 1
}

#growth function
gxy<-function(x,y) {
  mux<-g.inter+g.slope*x
  mux<-mux
  sigmax2<-v.growth
  sigmax<-sqrt(sigmax2)
  fac1<-sqrt(2*pi)*sigmax
  fac2<-((y-mux)^2)/(2*sigmax2)
  return(exp(-fac2)/fac1)
}

pxy<-function(x,y) { return(sx(x)*gxy(x,y)) }

#fecundity function
fxy<-function(x,y) {
  seeds<-f1.inter+f1.slope*x*seedn
  fac1<-sqrt(2*pi)*sqrt(var.sdl)
  fac2<-((y-mean.sdl)^2)/(2*var.sdl)
  f<-fx(x)*seeds*exp(-fac2)/fac1
  return(f)
}

#recruitment from the seed bank
recruit<-function(y){
  fac1<-sqrt(2*pi)*sqrt(var.sdl)
  fac2<-((y-mean.sdl)^2)/(2*var.sdl)
  f<-exp(-fac2)/fac1
  return(f)
}
```

```
#seed production
seedsx<-function(x) {
  seeds<-f1.inter+f1.slope*x*seedn
  f<-fx(x)*seeds
  return(f)
}

#################################################################

bigmatrix<-function(n) {
  # upper and lower integration limits
  L<-minsize;
  U<-maxsize;

  # boundary points b and mesh points y
  n<-n.big.matrix
  b<-L+c(0:n)*(U-L)/n
  y<-0.5*(b[1:n]+b[2:(n+1)])

  # construct the matrix
  I<-diag(n)
  M<-matrix(0,n,n)
  P<-matrix(0,n,n)
  B<-matrix(0,n,n)
  P<-t(outer(y,y,pxy))*(U-L)/n  #survival-growth matrix without sb

  ###############################################

  B<-t(outer(y,y,fxy))*(U-L)/n  #fecundity
  R<-recruit(y)*(U-L)/n         #seedlings per matrix class
  S<-seedsx(y)                  #seeds per matrix class

  return(list(Pmatrix=P,meshpts=y,Bmatrix=B,Smatrix=S,Rmatrix=R,Imatrix=I))
}   #for big matrix

################ Matrix transitions

Mt<-bigmatrix(n.big.matrix)
n<-n.big.matrix
M<-matrix(NA,nrow=n+1,ncol=n+1)  #add a row and column for the seed bank
M[2:(n+1),2:(n+1)]<-Mt$Pmatrix+e*Mt$Bmatrix   #transitions + new seedlings
M[1,2:(n+1)]<-(1-e)*Mt$Smatrix*ssb  #goes to the seed bank, 1. row
M[2:(n+1), 1]<-Mt$Rmatrix*esb      #recruitment from the seed bank
M[1,1]<-ssb                        #constant seed survival in the seed bank
lam<-lambda(M)   #population growth rate (needs popbio package)

############################
# Check eviction: plot the survival model against the column sums of
survival-growth matrix
su<-(s.inter+s.slope*log(popdata$Size.t))
sur<-(exp(su)/(1+exp(su)))
plot(log(popdata$Size.t)[!is.na(popdata$Size.t)],
sur[!is.na(popdata$Size.t)], type="l", ylim=c(0, 1), xlim=c(2,5),
ylab="Survival")
points(Mt$meshpts, colSums(Mt$Pmatrix))

# Check eviction: plot the predicted growth values against observed ones
gr<-(g.inter+g.slope*log(popdata$Size.t))
plot(log(popdata$Size.t)[!is.na(popdata$Size.t)],gr[!is.na(popdata$Size.t)]
, type="l", ylim=c(2, 4.5), xlim=c(2,4.5), ylab="Growth")
points(log(popdata$Size.t), log(popdata$Size.t1))
```
